# Meteorological Table

**Published:** 1852-03

**Authors:** Lawrence Young


### METEOROLOGICAL TABLE,

### FOR THE MONTH OF JANUARY, 1852.

By Lawrence Young, Esq.

| Day of month, . . . . | Morning, . . . . . . | 2 o'clock, P. M., . . . | Evening, . . . . . . . | Mean temperature, . . | Barometer, . . . . . . . | Rain-guage, . . . . . . . | Course of wind, . . . . | Remarks. |
|---|---|---|---|---|---|---|---|---|
| 1 | 34 | 37 | 36 | 36 | 29.27 | | n. | Cloudy. |
| 2 | 32 | 34 | 32 | 33 | 29.28 | | n. w. | Do. |
| 3 | 29 | 33 | 31 | 31 | 29.17 | | n. n. w. | Do. |
| 4 | 26 | 41 | 38 | 35 | 29.10 | | s. s. w. | Variable. |
| 5 | 38 | 34 | 32 | 35 | 28.77 | 61† | w. | Cloudy. |
| 6 | 29 | 29 | 27 | 29 | 28.88 | 11‡ | w. | Do. |
| 7 | 24 | 26 | 25 | 25 | 29.22 | | w. s. w. | Do. |
| 8 | 22 | 32 | 32 | 29 | 28.99 | 10‡ | s. | Do. |
| 9 | 28 | 32 | 28 | 29 | 28.84 | 4‡ | w. s. w. | Do. |
| 10 | 26 | 32 | 30 | 29 | 28.94 | | n. n. w. | Do. |
| 11 | 23 | 25 | 19 | 22 | 29.16 | 24‡ | n. n. w. | Do. |
| 12 | 0 | 14 | 7 | 7 | 29.54 | | w. | Clear. |
| 13 | –4 | 16 | 14 | 11 | 29.44 | | swly. | Do. |
| 14 | 13 | 36 | 32 | 27 | 29.08 | | s. s. w. | Do. |
| 15 | 23 | 35 | 32 | 30 | 28.91 | | w. s. w. | Variable. |
| 16 | 32 | 39 | 33 | 35 | 29.— | | wly. | Do. |
| 17 | 20 | 39 | 30 | 30 | 29.24 | | e. n. e. | Do. |
| 18 | 20 | 18 | 17 | 18 | 29.14 | 41‡ | n. n. w. | Cloudy. |
| 19 | –11 | –2 | –4 | –5 | 29.56 | | w. | Clear. |
| 20 | –7 | 15 | 11 | –6 | 29.50 | | s. s. w. | Variable. |
| 21 | 15 | 24 | 18 | 19 | 29.19 | 6‡ | w. s. w. | Cloudy. |
| 22 | –4 | 20 | 13 | 10 | 29.54 | | s. w. | Clear. |
| 23 | 5 | 32 | 24 | 20 | 29.55 | | s. w. | Do. |
| 24 | 9 | 42 | 30 | 27 | 29.51 | | s. w. | Do. |
| 25 | 14 | 44 | 34 | 31 | 29.32 | | w. s. w. | Variable. |
| 26 | 33 | 40 | 30 | 34 | 29.15 | 12‡ | w. s. w. | Do. |
| 27 | 15 | 39 | 30 | 28 | 29.19 | | s. s. w. | Clear. |
| 28 | 32 | 51 | 43 | 42 | 29.07 | | s. w. | Variable. |
| 29 | 33 | 57 | 44 | 45 | 29.05 | | w. s. w. | Clear. |
| 30 | 48 | 61 | 51 | 53 | 29.08 | | s. s. w. | Variable. |
| 31 | 52 | 54 | 46 | 51 | 28.94 | 12§ | n. n. w. | Do. |
| | | | | 27.4 | | 1.81 | | |

† This sign denotes snow and rain. ‡ Snow. § Rain.

On the 19th, at 12 o'clock, M., the thermometer stood at 18 degrees below zero, which was the period of most intense cold.

